# CSF-diverting shunts: Implications for abdominal and pelvic surgeons; a review and pragmatic overview

**DOI:** 10.1016/j.amsu.2019.10.033

**Published:** 2019-11-05

**Authors:** Aimee Goel, Claudia Craven, Samir Matloob, Simon Thompson, Laurence Watkins, Ahmed Toma

**Affiliations:** Victor Horsley Department of Neurosurgery, National Hospital for Neurology and Neurosurgery, University College London Hospitals NHS Foundation Trust, London, WC1N 3BG, UK

**Keywords:** CSF-Diverting shunt, Shunt, Neurosurgery, Abdominal surgery, Pelvic surgery

## Abstract

The optimal management of patients with ventriculoperitoneal or lumboperitoneal shunts undergoing abdominal or pelvic surgery for unrelated reasons is often unclear due to the paucity of guidelines in this field. In this review, we outline key issues in managing these patients. Specifically, we address issues relating to pre-operative planning, avoidance of shunt-related complications such as infection and malfunction, and specific management of neurological symptoms in the post-operative period.

A retrospective study was carried out analysing correspondence between general surgeons and a specialist hydrocephalus unit over a 4-year period relating to management of patients with ventriculoperitoneal and lumboperitoneal shunts undergoing abdominal or pelvic surgery. A literature review was carried out to identify available evidence in this field. 30 queries from general surgeons were identified comprising 12 main themes. 16 relevant publications were identified. We summarised these to answer these queries.

The management of shunted patients may present challenges and uncertainties in an abdominal or pelvic surgery setting. This paper provides guidelines and clarity in this field by discussing and summarising reported data in the literature.

## Highlights

• The optimal management of VP or LP shunted patients undergoing abdomen/pelvic surgery is unclear.
• We provide guidelines for surgeons in this field by summarising the reported data.
• We cover pre-operative, peri-operative and post-operative considerations in these patients.

## 1 Introduction

The National Hospital for Neurology and Neurosurgery in London is a quaternary neurosurgical unit with a specialist hydrocephalus service. As a national referral unit, the hydrocephalus team receives inquiries from gastrointestinal, colorectal, urological and general surgeons from 12 hospitals in London requesting specialist advice regarding patients with CSF-diverting shunts undergoing laparoscopic or open abdominal or pelvic surgery. These frequently relate to pre-operative precautions, peri-operative considerations in shunt handling, and post-operative management of shunt-related complications. Such referrals are logged and accessed in an online database, categorising referrals by question. This database provides a useful tool for retrospective analysis of common questions regarding shunts in the context of general surgery.

There is a paucity of evidence relating to management of shunted patients undergoing abdominal or pelvic surgery and measures to avoid shunt-related complications. To our knowledge, there are no articles with comprehensive evidence-based recommendations aimed at abdominal or pelvic surgeons operating in these patients. This article aims to provide a pragmatic overview of commonly queried pre-operative, peri-operative and post-operative considerations in these patients.

## 2 Methods

A retrospective analysis of the correspondence between abdominal/pelvic surgeons and our hydrocephalus unit was performed to identify common queries about shunt management. We reviewed all shunt-related queries posted on a common web-based referral system to our unit between 2014 and 2018. Responses to questions were based on available literature in this field as well as specialist clinical experience of the Hydrocephalus team.

A PubMed literature search was performed relating to the complications of abdominal surgery in patients with CSF-diverting shunts using the MeSH terms ‘ventriculoperitoneal shunt’, ‘lumboperitoneal shunt’, ‘CSF-diverting shunt’, ‘Laparoscopic surgery’, ‘laparoscopy’, ‘abdominal surgery’, ‘complications’, ‘infection’. We excluded papers relating to abdominal surgery for shunt-related complications, and complications of shunts more broadly.

## 3 Results

A total of 30 queries from general surgeons were received between 2014 and 2018 relating to either laparoscopic or open surgery in patients with either ventriculoperitoneal (VP) or lumboperitoneal (LP) shunts. Of these, 12 main referral themes were identified. These were subdivided into those relating to pre-operative, peri-operative or post-operative management (Table 1).

**Table 1:** Some common considerations with regards to shunt management in abdominal and pelvic surgery.

1. Pre-operative	1.1 Determining shunt location
	1.2 Shunt MRI safety
	1.3 Antibiotic prophylaxis
2. Peri-operative	2.1 Normal findings in shunted patients during abdominal surgery
	2.2 Increased abdominal pressure and shunt function
	2.3 Considerations in clean abdominal surgery
	2.4 Shunt protection in extended/dirty abdominal surgery
	2.5 Surgical technique
3. Post-operative	3.1 Management of post-operative neurological symptoms
	3.2 Management of post-operative pain
	3.3 Abdominal Pain
	3.4 Effect of adhesions on shunt

A total of 531 papers were identified during literature search, out of which 16 were selected as being relevant following a review of titles and abstracts. These primarily included case series, reviews of case series and case reports. In the discussion, we proceed to answer the above 12 queries based on evidence obtained from the literature search.

## 4 Discussion

### 4.1 Pre-operative considerations

#### 4.1.1 Determining shunt location

**Fig. 1:**
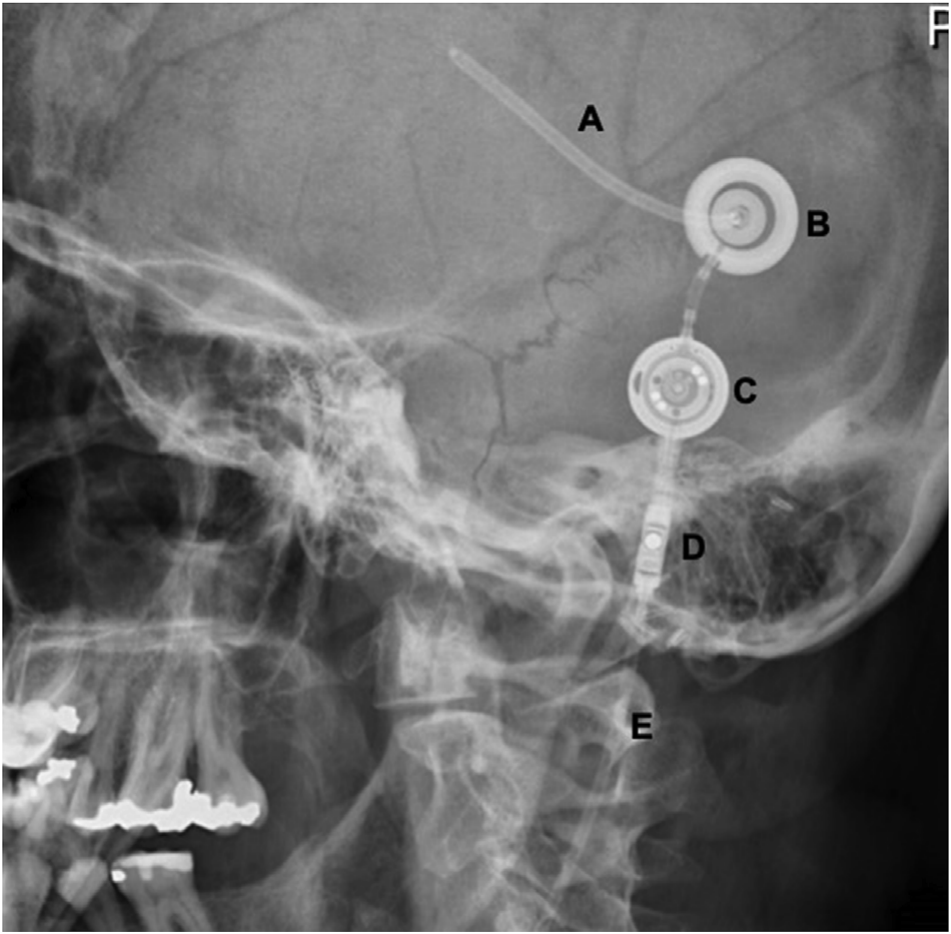
Lateral skull X-ray confirming a ventricular shunt including the proximal ventricular catheter (A), reservoir (B), adjustable valve (C), gravitation unit (D), and the distal catheter tunnelled subcutaneously (E).

**Fig. 2:**
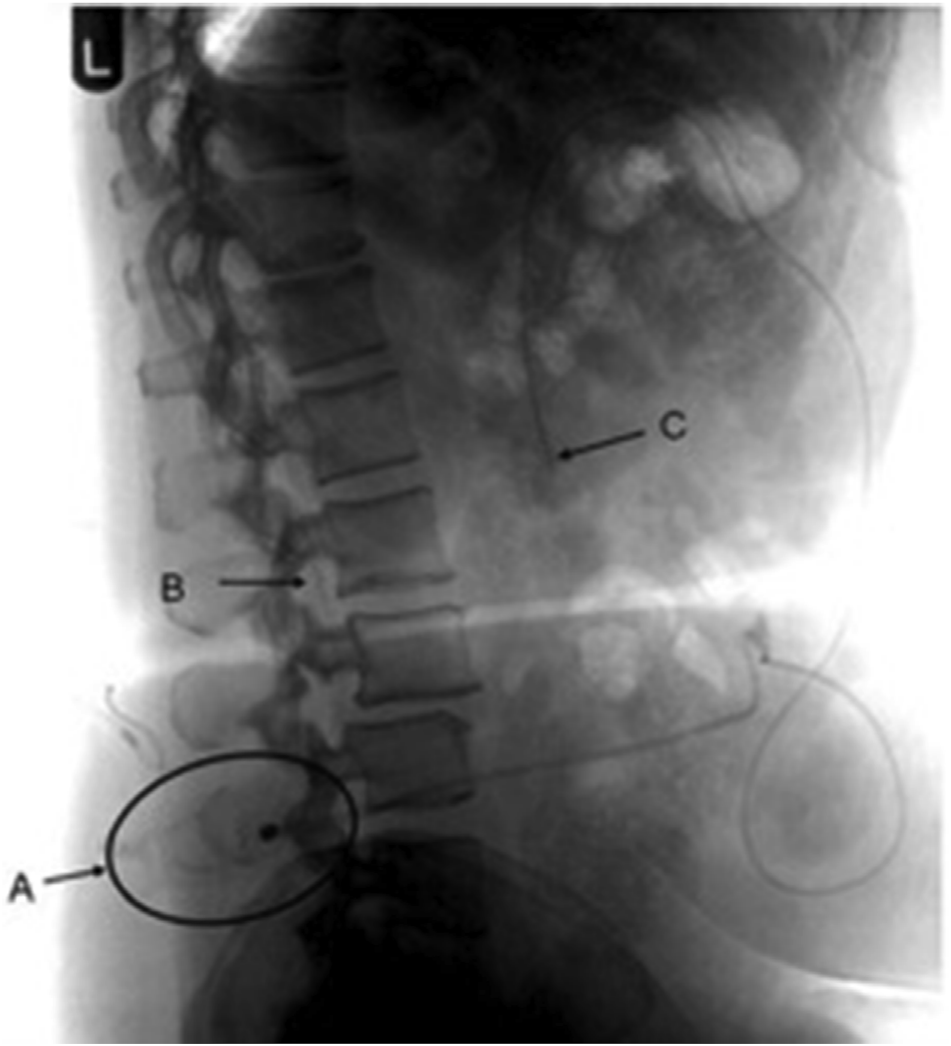
Lateral abdominal radiograph confirming an LP shunt with its proximal catheter within the spinal canal (B), a valve (A), and the distal catheter tip (C) lying intraperitoneally. Figure adapted from Toma et al., 2010 [2].

The location of the tip of the shunt as well as the presence or absence of a valve are relevant considerations during abdominal surgery. Lack of awareness of the shunt tip may lead to accidental shunt transection or damage, while lack of awareness of the shunt valve may affect intraoperative CSF drainage [1]. The proximal end of the VP shunt is placed in the lateral ventricle and tunnelled subcutaneously. The distal end is placed intraperitoneally via a small abdominal incision. With regards to LP shunts, the proximal catheter is placed in the thecal sac at the L3/4 level, and the distal catheter into the peritoneal cavity. The vast majority of VP and LP shunts have valves regulating the pressure gradient and amount of CSF drained. The presence of a valve can be confirmed with a plain lateral head X-ray for VP shunts (Fig. 1), or with an abdominal X-ray for LP shunts (Fig. 2).

**Fig. 3:**
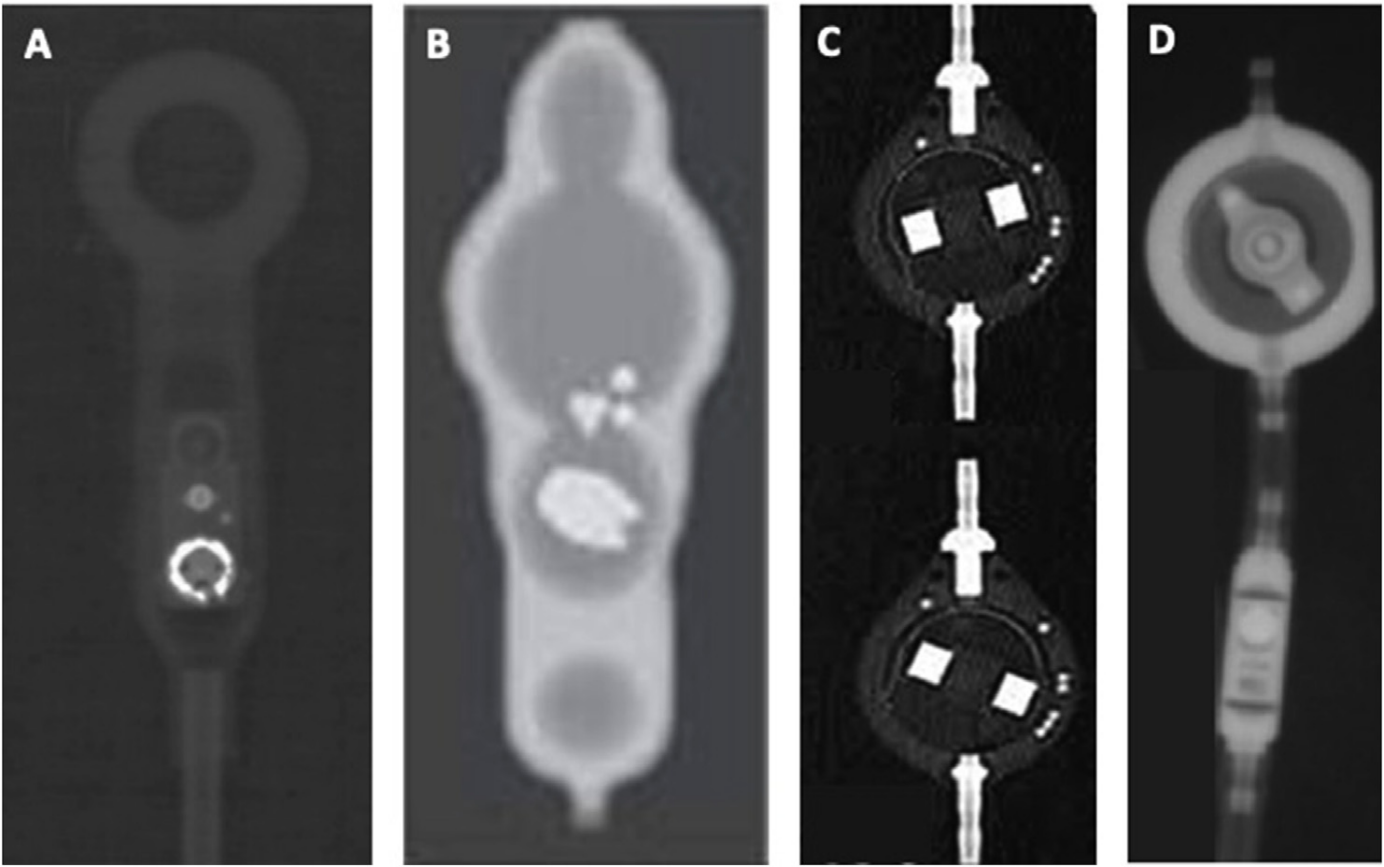
Radiographic appearances of some commonly used shunt valve systems, Codman Hakim (A), Medtronic Strata (B), Sophysa Sophy SM6 (C), Aesculap Miethke ProGAV (D). Figures adapted from Lollis et al., 2010 and Handbook of Neurosurgery, Greenberg, 2010 [3,4].

Shunt valves have many different appearances. If uncertainty exists with regards to valve type or location, a neurosurgical team should be contacted. Fig. 3 depicts radiographic appearances of some commonly used shunt valves.

#### 4.1.2 Shunt MRI safety

The majority of shunts are safe and MRI compatible (up to 3 T) however certain valve types may require re-programming following MRI. Fixed or ‘non-adjustable’ valves permit CSF drainage of either a particular amount or if exceeding a particular pressure. Fixed valves are non-reprogrammable and therefore safe for MRI scanning. However, adjustable valves adjusted to various CSF pressure or flow settings are more commonly used. Such valves are adjusted transcutaneously using a specialised tool and a coded magnetic field [3]. Some of these valves may therefore undergo readjustment in an MRI scanner; this is manufacturer dependent. The patient's shunt identification card contains details regarding MRI safety, along with a contact number for the patient's hydrocephalus team or specialist nurse, who should be contacted in cases of uncertainty. Failing that, the neurosurgery on-call team should be able to advise if the valve is one that requires re-programming.

#### 4.1.3 Antibiotic prophylaxis

The literature review revealed a very low incidence of shunt-related CNS infection or peritonitis following abdominal surgery. In most cases, no special precautions were taken to prevent infection. A retrospective review of 39 abdominal and urological operations showed minimal risk for infection over a 2–10 year follow up period in patients undergoing routine clean or clean-contaminated laparoscopic or open surgery [5]. The duration and type of antibiotic prophylaxis varied, from no antibiotic prophylaxis, to several days of prophylactic antibiotics. The authors recommended shunt externalisation in dirty surgeries with purulent material present. Similarly, a retrospective review of 25 children with VP shunts undergoing gastrostomy tube placement showed a similar post-operative course and no higher risk of complications within a 90-day follow up period compared to non-shunted children undergoing the same procedure [6]. Two retrospective studies into patients undergoing abdominal surgery showed a low incidence of post-operative shunt infection in two patients each – these patients had undergone emergency surgery; either ‘dirty’ surgery (appendectomies for peritonitis), or clean-contaminated surgery [7,8]. Shunt infection occurred immediately post-operatively for the dirty surgeries, and 4–6 months later for the clean-contaminated surgeries.

In our experience, we have not come across shunt related infection in a patient who has undergone elective clean or clean-contaminated abdominal or pelvic surgery. We do not recommend any special perioperative antibiotic prophylaxis or special shunt manipulation in these patients. However, in emergency ‘dirty’ or contaminated surgery, the neurosurgery team should be contacted as the shunt may require prior removal or externalisation, and the local microbiology team should be consulted regarding antibiotics to cover for CNS infection. Further studies comparing antibiotic prophylaxis and shunt-infection outcomes are warranted.

### 4.2 Intra-operative considerations

#### 4.2.1 Expected peri-operative findings in shunted patients

A small amount of colourless CSF is often present within the peritoneum in shunted patients and may be visible on scans or intraoperatively. This is not a cause for concern and is usually reassurance of normal shunt function. Very rarely, pseudocysts around the tip of the distal catheter may be visible on imaging or intra-operatively. These usually present with abdominal pain and distension but may be asymptomatic in the initial stages [9]. If encountered, these cysts may be drained, and the distal catheter repositioned within the peritoneum away from the cyst.

#### 4.2.2 Abdominal pressure in laparoscopic surgery and shunt function

Increased intra-abdominal pressure may lead to increased intracranial pressure (ICP) mainly due to associated increased intrathoracic pressure and impaired lumbar plexus venous drainage. Physiological intraabdominal pressure fluctuates continually from 20 mmHg while standing to 81.4 mmHg while coughing [10]. In comparison, gas insufflation during laparoscopic surgery results in an increase in intra-abdominal pressure to only 10–20 mmHg [11]. In vitro studies have shown no retrograde CSF flow in shunts even with pressures as high as 350 mmHg [12]. In 51 paediatric patients with VP shunts undergoing laparoscopic surgery, no episodes of air embolus were found [13]. A review of 7 studies of laparoscopic cholecystectomy in CSF shunts with pressures up to 15 mmHg, no symptoms of shunt malfunction were reported post-operatively [14]. Further, no differences in outcome were reported for shunts that were clamped versus unclamped during laparoscopic surgery [14]. Similarly, for LP-shunted patients undergoing laparoscopic surgery, no intraoperative or immediate post-operative neurological changes associated with raised ICP have been noted [15].

In conclusion, in the presence of a working unidirectional valve, minor increases in intra-abdominal pressure during laparoscopic surgery should not cause CSF back flow. Major or prolonged increases in intra-abdominal pressure could, in theory, result in a raised ICP in patients who are extremely shunt dependent. We however do not routinely recommend shunt clamping if the shunt is to remain internalised in short and routine laparoscopic procedures.

#### 4.2.3 Clean abdominal surgery

For clean abdominal surgery where the distal end of the shunt tube will be encountered, the distal end can be clamped with a guarded artery clip and wrapped in betadine gauze away from the surgical field, and then reinserted prior to closure. During closure, one should ensure that the tube is placed intra-peritoneally and that all defects in the peritoneum wall and muscular layers are closed.

#### 4.2.4 Extended or ‘dirty’ abdominal surgery

There are no studies evaluating methods of externalisation or comparative outcomes in externalised versus non-externalised shunts for ‘dirty’ surgery. A common and safe method is to externalise the drain. The rationale for this approach is to reduce the chances of shunt infection while maintaining CSF drainage in shunt dependant hydrocephalus patients. We have summarised the stages below:

1. Externalise the peritoneal end of the shunt tube through a separate incision away from any other abdominal drains.
2. CSF samples may be taken from the distal end of the tubing using sterile technique, for routine microbiological testing.
3. A sterile dressing should be applied to the skin exit point to avoid direct tube contact with skin.
4. The shunt tube should be shortened and connected to a specialised CSF drainage bag (eg. Becker's drain) using a connector, as in Fig. 4.

**Fig. 4:**
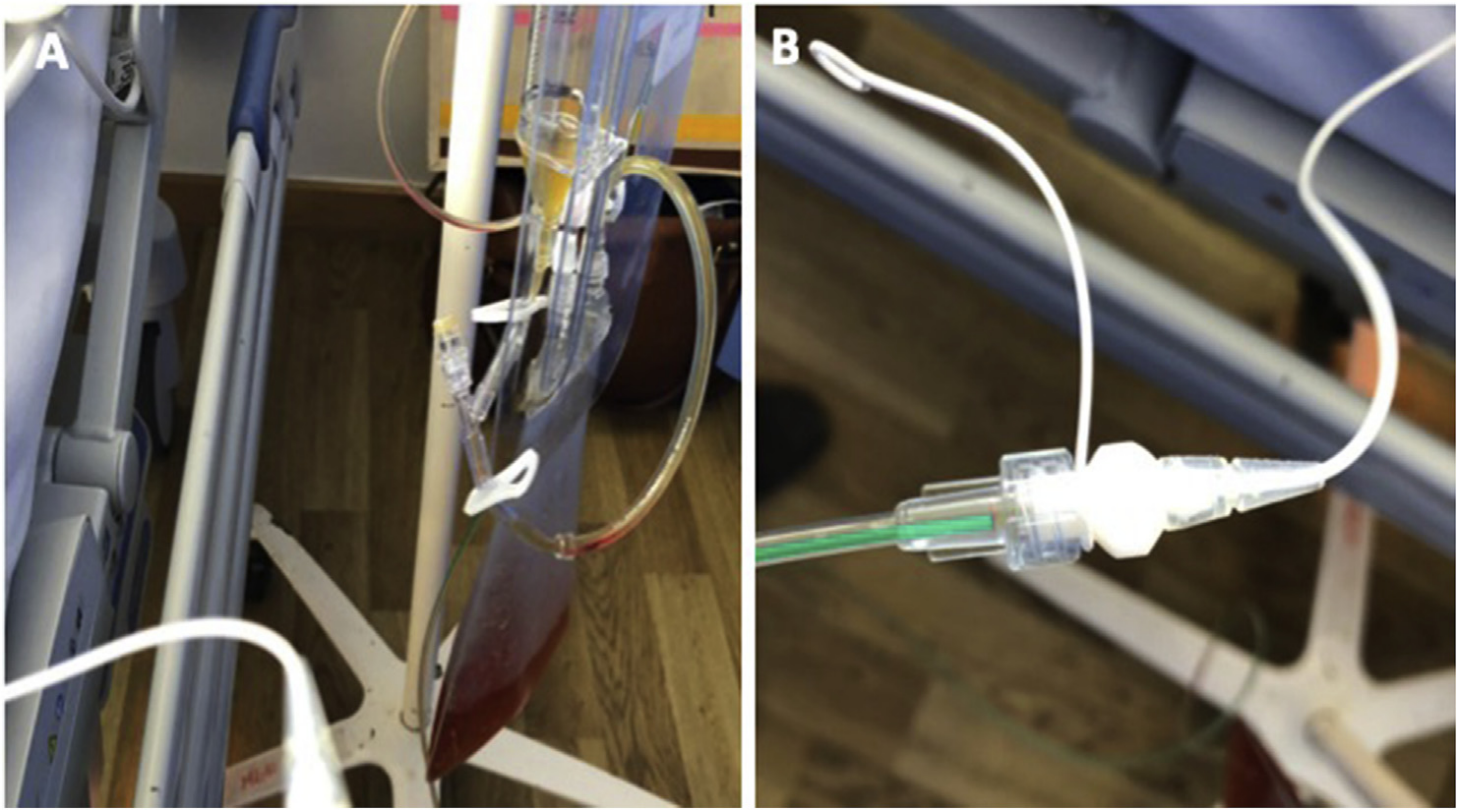
An externalised shortened shunt connected to a Becker drain set (image A) using a connector (image B) and placed level with the abdomen.
5. The drain should be closed during patient transfer and operative positioning of the patient. It is important to ensure it is re-opened after transfer and positioning, to prevent acute hydrocephalus. During surgery, continuous drainage should be carried out rather than repeated clamping and unclamping.
6. The bag should be kept at the level of the abdomen. Dropping the drain to the floor may rapidly empty the patient's ventricles of CSF if no valve is present. Placing the drain above this level will reduce CSF drainage.
7. After surgery the drain should remain externalised. CSF drainage should be charted hourly on the fluid input/output chart, and the drain should be clamped temporarily if extreme over-drainage is recorded.
8. Antibiotic treatment should be guided by local guidelines and the nature of the procedure.
9. Subsequent liaison with the neurosurgeon is needed to agree on future shunt management. This will depend on the patient's general status. Patients may need an alternative destination for the distal catheter in the form of ventriculoatrial or ventriculopleural shunts.

#### 4.2.5 Surgical technique

Diathermy, both monopolar and bipolar, can be used routinely in patients with shunts, including those with metallic valves.

### 4.3 Post-operative considerations

#### 4.3.1 New post-operative neurological symptoms

**Fig. 5:**
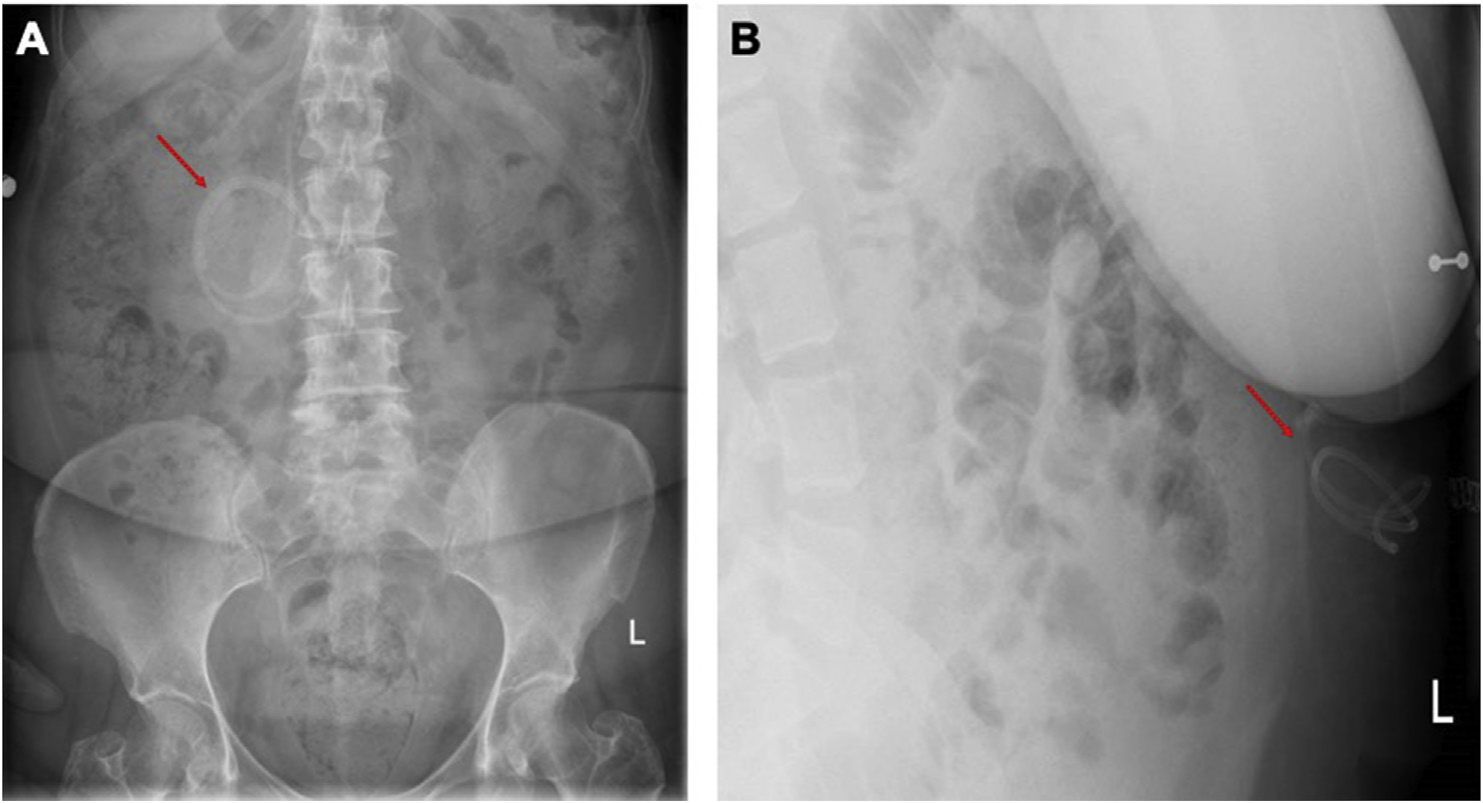
Abdominal X-rays in AP (A) and lateral (B) views in different patients showing incorrectly positioned extra-peritoneal VP shunt tubing.

Post-operative neurological complications are rare following abdominal or pelvic surgery in shunted patients, however symptoms of shunt dysfunction (eg. headache, increasing drowsiness or vomiting) or CNS infection (eg. fever, neck stiffness) warrants a contrast-enhanced CT head to investigate for new ventriculomegaly or infection. A plain abdominal film is also indicated looking for sub-optimal replacement of the distal catheter, such as extra-peritoneal placement demonstrated in Fig. 5. New onset radicular pain post operatively in patients with LP shunts may indicate catheter migration and should be investigated with an AP and lateral abdominal radiograph and a spinal radiograph in the first instance. Discussion with a local neurosurgery unit is necessary to determine the need for shunt replacement.

#### 4.3.2 Post-operative pain

Post-operative pain in shunted patients should be managed in a similar way to non-shunted patients. In patients prone to analgesia overuse headaches such as those with idiopathic intracranial hypertension, we recommend avoiding prolonged courses of opioids [16].

#### 4.3.3 Abdominal pain primarily due to a shunt

Abdominal pain can occur due to shunt tubing. The aetiology for this is poorly understood but there are case reports suggesting that tube length and distal tip location could play a role. Further, there is anecdotal evidence suggesting that some shunt material types could be more irritant than others, however this is an area that requires further research [[17], [18], [19]]. The likelihood of abdominal pain being due to a general surgical, urological or gynaecological cause is far greater however, and these should be explored in the first instance. If all these causes are ruled out, the patient may require a trial of shunt externalisation in a hydrocephalus unit, to test for improvement of abdominal pain.

#### 4.3.4 Adhesions

Adhesions following repeated abdominal surgeries in shunted patients could lead to shunt constriction or displacement and impaired drainage. This may present with headache, drowsiness and nausea. If widespread adhesions are noted during abdominal surgery in a shunted patient who is symptomatic for shunt obstruction, discussion with a neurosurgical unit is warranted for the consideration of distal catheter relocation to a different drainage site.

## 5 Conclusion

There is a lack of high-powered and randomized controlled studies to provide a clear evidence base with reagards to management of shunted patients within an abdominal and pelvic surgery setting. Further studies comparing outcomes in externalised versus non-externalised shunts, open versus laparoscopic approaches, and in patients that receive special versus no special antibiotic prophylaxis are warranted. These studies are even more clinically relevant due to increasing numbers of shunted patients with idiopathic intracranial hypertension undergoing bariatric surgery.

The shunted patient can present challenges in a general surgery setting, and lack of experience with CSF drainage devices may lead to uncertainty when operating in these patients. This review should answer some common questions relating to abdominal and pelvic surgery in shunted patients. It is important for surgeons, and for patients to recognise the signs and symptoms of the unlikely but potential shunt-related complications post-surgery. In more complicated cases, for example in patients with previous or current shunt-related abdominal complications, it is advisable to seek opinion from a specialist hydrocephalus unit regarding precautions prior to surgery.

## Provenance and peer review

Not commissioned, externally peer reviewed.

## Ethical approval

Not applicable.

## Sources of funding

No funding was received to conduct this study.

AT research time was supported by the National Institute for Health Research,

University College Hospitals Biomedical Research Centre.

## Author contribution

AG: Writing manuscript content, revisions.

CC: Addition to manuscript content, revisions.

SM: Manuscript initial design.

ST: Input into manuscript concept.

LW: Concept.

AT: Concept, review of drafts and content revision, approval of final manuscript.

## Consent

Not applicable.

## Registration of research studies

Not applicable.

## Guarantor

Ahmed Toma.

## Declaration of competing interest

No conflicts of interest.
